# MNS1 Is Essential for Spermiogenesis and Motile Ciliary Functions in Mice

**DOI:** 10.1371/journal.pgen.1002516

**Published:** 2012-03-01

**Authors:** Jian Zhou, Fang Yang, N. Adrian Leu, P. Jeremy Wang

**Affiliations:** Department of Animal Biology, Center for Animal Transgenesis and Germ Cell Research, University of Pennsylvania School of Veterinary Medicine, Philadelphia, Pennsylvania, United States of America; Washington University School of Medicine, United States of America

## Abstract

During spermiogenesis, haploid round spermatids undergo dramatic cell differentiation and morphogenesis to give rise to mature spermatozoa for fertilization, including nuclear elongation, chromatin remodeling, acrosome formation, and development of flagella. The molecular mechanisms underlining these fundamental processes remain poorly understood. Here, we report that MNS1, a coiled-coil protein of unknown function, is essential for spermiogenesis. We find that MNS1 is expressed in the germ cells in the testes and localizes to sperm flagella in a detergent-resistant manner, indicating that it is an integral component of flagella. MNS1–deficient males are sterile, as they exhibit a sharp reduction in sperm production and the remnant sperm are immotile with abnormal short tails. In MNS1–deficient sperm flagella, the characteristic arrangement of “9+2” microtubules and outer dense fibers are completely disrupted. In addition, MNS1–deficient mice display situs inversus and hydrocephalus. MNS1–deficient tracheal motile cilia lack some outer dynein arms in the axoneme. Moreover, MNS1 monomers interact with each other and are able to form polymers in cultured somatic cells. These results demonstrate that MNS1 is essential for spermiogenesis, the assembly of sperm flagella, and motile ciliary functions.

## Author Summary

Cilia are microtubule-based structures present in virtually all cells in vertebrates. Cilia have diverse functions in development, growth, signaling, and fertilization. Primary ciliary dyskinesia (PCD) affects one in 16,000 individuals. PCD is characterized by bronchiectasis and chronic sinusitis, and is often associated with situs inversus and male infertility. The genetic cause of PCD is heterogeneous. Some cases of PCD in humans and animals are caused by single genic mutations such as mutations in genes encoding microtubule-based dynein arm components. We have characterized a protein called MNS1 and found that it plays an essential role in ciliary functions in mice. MNS1 is a novel and integral component of sperm flagella. Mice lacking MNS1 exhibit male sterility as evidenced by abnormal assembly of sperm flagella. MNS1–deficient mice also display defects in left–right asymmetry patterning of internal organs and hydrocephalus. Therefore, mutations in MNS1 may contribute to male infertility and PCD in humans.

## Introduction

Spermatogenesis is divided into three phases: mitotic, meiotic and haploid. During the haploid phase (spermiogenesis), spermatids undergo a complex differentiation process to develop into spermatozoa, including chromatin remodeling, nuclear elongation, cytoplasm elimination, acrosome formation, and flagellum development [1]. The sperm flagellum is a complex structure whose integrity is essential for sperm motility and fertilization of the egg [2], [3]. Structural defects in the flagella of sperm from infertile men are responsible for motility abnormalities and may underlie some cases of male infertility in humans [4].

In contrast with flagella, cilia are present in nearly all cell types in vertebrate and play diverse functions [5], [6]. For example, respiratory cilia are important for mucus clearance [7]. Ependymal cilia facilitate cerebrospinal fluid flow [8]–[10]. Nodal cilia are essential for left-right asymmetry patterning during embryogenesis [11], [12]. Flagella and some motile cilia such as respiratory and ependymal cilia consist of nine outer doublet microtubules and one pair of single microtubules in the center (9+2 axoneme). Nodal cilia lack the central pair of microtubules and thus contain a 9+0 axoneme. In the axoneme of motile cilia, dynein arms, ATP-dependent motor proteins, are attached to and projected from outer doublet microtubules. Primary ciliary dyskinesia (PCD) is a heterogeneous group of autosomal recessive disorders characterized by recurrent respiratory infections [13]. Half of PCD patients display situs inversus and PCD is sometimes associated with male infertility. Flagella and cilia are complex structures that consist of >600 proteins [5]. Genetic studies in diverse organisms have begun to uncover the role for some of these proteins in ciliary and flagellar functions [9], [10], .

Mouse MNS1 (meiosis-specific nuclear structural protein 1) was previously identified due to cross-reactivity with an anti-lamin antibody in a study of the perinuclear matrix [20]. In this original investigation, MNS1 appeared to be expressed specifically in testes. The MNS1 protein contains long coiled-coil domains but no other known functional motifs. In that report, it was concluded that MNS1 was specifically expressed in pachytene spermatocytes and thus might function in maintaining proper nuclear morphology during meiosis [20]. However, the physiological function of MNS1 remains unknown. Here we report that, in contrast with the previous study [20], MNS1 is abundantly expressed in post-meiotic spermatids and is required for spermiogenesis. In addition, MNS1 is also required for motile ciliary functions.

## Results

### MNS1, a Coiled-Coil Protein, Is an Integral Component of Sperm Flagella

**Figure 1:**
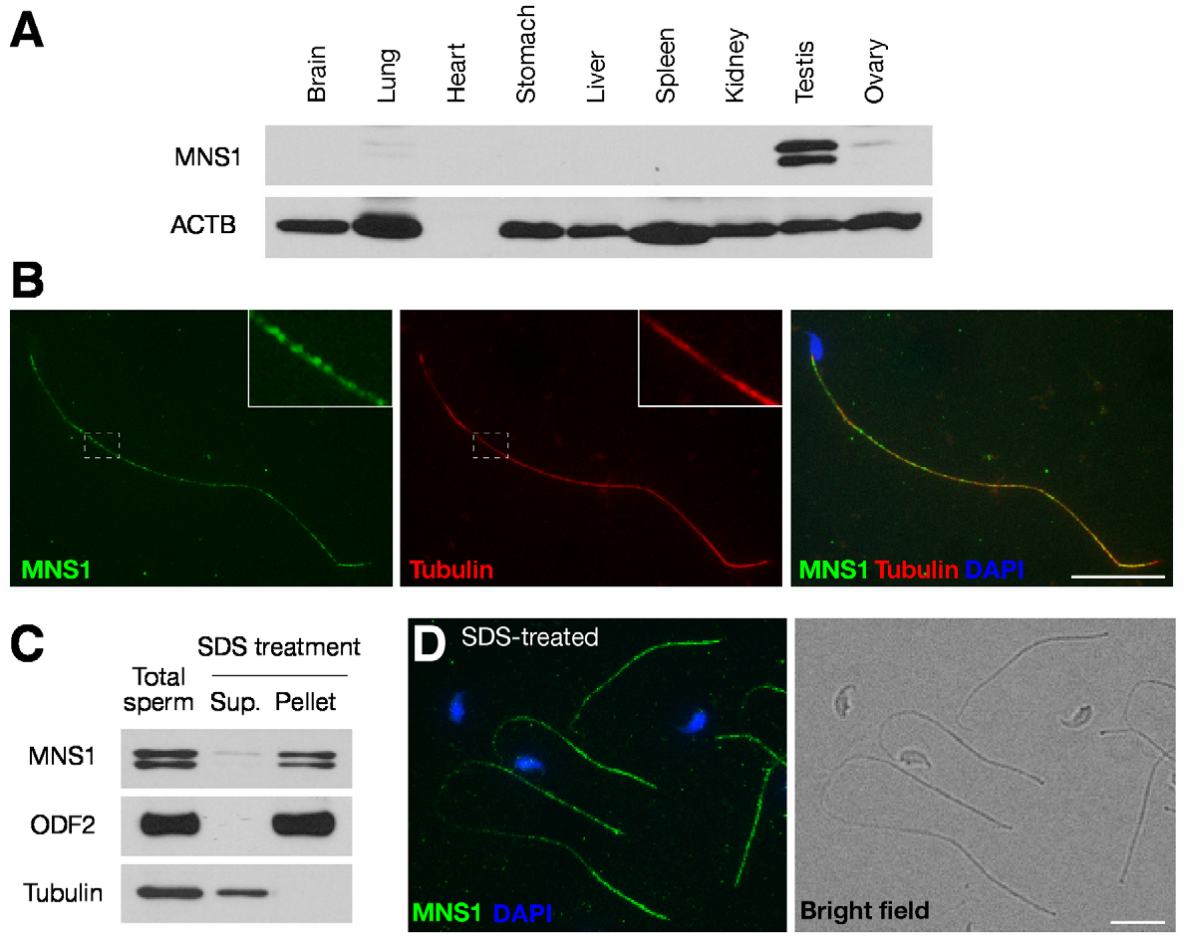
MNS1 is an integral component of sperm flagella. (A) Western blot analysis of MNS1 in adult mouse tissues. Anti-MNS1 antibody UP2060 was used. ACTB served as a control. Note that heart contains little ACTB. (B) Immunostaining of cauda epididymal sperm with anti-MNS1 (UP2284) and anti-tubulin antibodies. (C) Western blot analysis of soluble (Sup.) and SDS-resistant (Pellet) sperm flagellar proteins using anti-MNS1 antibody (UP2060). Tubulin (Sup.) and ODF2 (Pellet) served as controls [22]. (D) Immunostaining of SDS-treated sperm flagella with anti-MNS1 antibody UP2060 (left). Shown on the right is the bright-field image. Scale bars. 25 µm.

To study the expression and localization of MNS1, we generated specific antibodies against the N-terminal and C-terminal fragments of mouse MNS1 respectively. Western blot analysis of adult mouse tissues revealed that MNS1 is abundantly expressed as two closely migrating protein species in the testis but not in other tissues except at very low levels in lung and ovary (Figure 1A). Immunofluorescence analysis showed that the expression of MNS1 is restricted to germ cells in the testis and appears to be predominantly cytoplasmic (Figure S1). The MNS1 protein was first detected at a low level in pachytene spermatocytes at stage VIII and then was abundantly expressed in late pachytene spermatocytes, diplotene spermatocytes, and post-meiotic spermatids (Figure S1). MNS1 was not detected in early germ cells ranging from spermatogonia to mid-pachytene spermatocytes (Figure S1). Our results showed that the expression of MNS1 is not restricted to pachytene spermatocytes as had been previously reported [20].

Immunofluorescence analysis showed that MNS1 localizes to sperm flagella (Figure 1B). In contrast with the relatively continuous distribution of tubulin in the sperm tail, MNS1 localization was not uniform, appearing in a dot-on-a-string fashion (Figure 1B, inset). Our MNS1 antibody was specific, since no staining was observed with *Mns1*-deficient sperm (data not shown). To assess the biochemical nature of MNS1 localization in sperm tails, we treated epididymal sperm with SDS-EDTA solution to divide sperm proteins into two fractions: soluble (supernatant) and SDS-resistant (pellet) (Figure 1C) [21], [22]. While SDS-treatment solublizes the tubulin components of the axoneme, other axonemal components (such as Tektin), outer dense fibers (ODF), mitochondrial sheath (MS), and fibrous sheath (FS) remain in the SDS-resistant structures [22], [23]. Western blot analysis showed that MNS1, like ODF2, resides in the SDS-resistant flagellar structures (Figure 1C). This result was consistent with the identification of MNS1 as one of the SDS-resistant proteins in the sperm flagella in a systematic proteomic profiling study [22]. As expected, MNS1 still localizes to SDS-treated sperm flagellar remnants (Figure 1D). Collectively, these data demonstrate that MNS1 is an integral component of sperm flagella.

### MNS1 Is Essential for Spermiogenesis and Male Fertility

**Figure 2:**
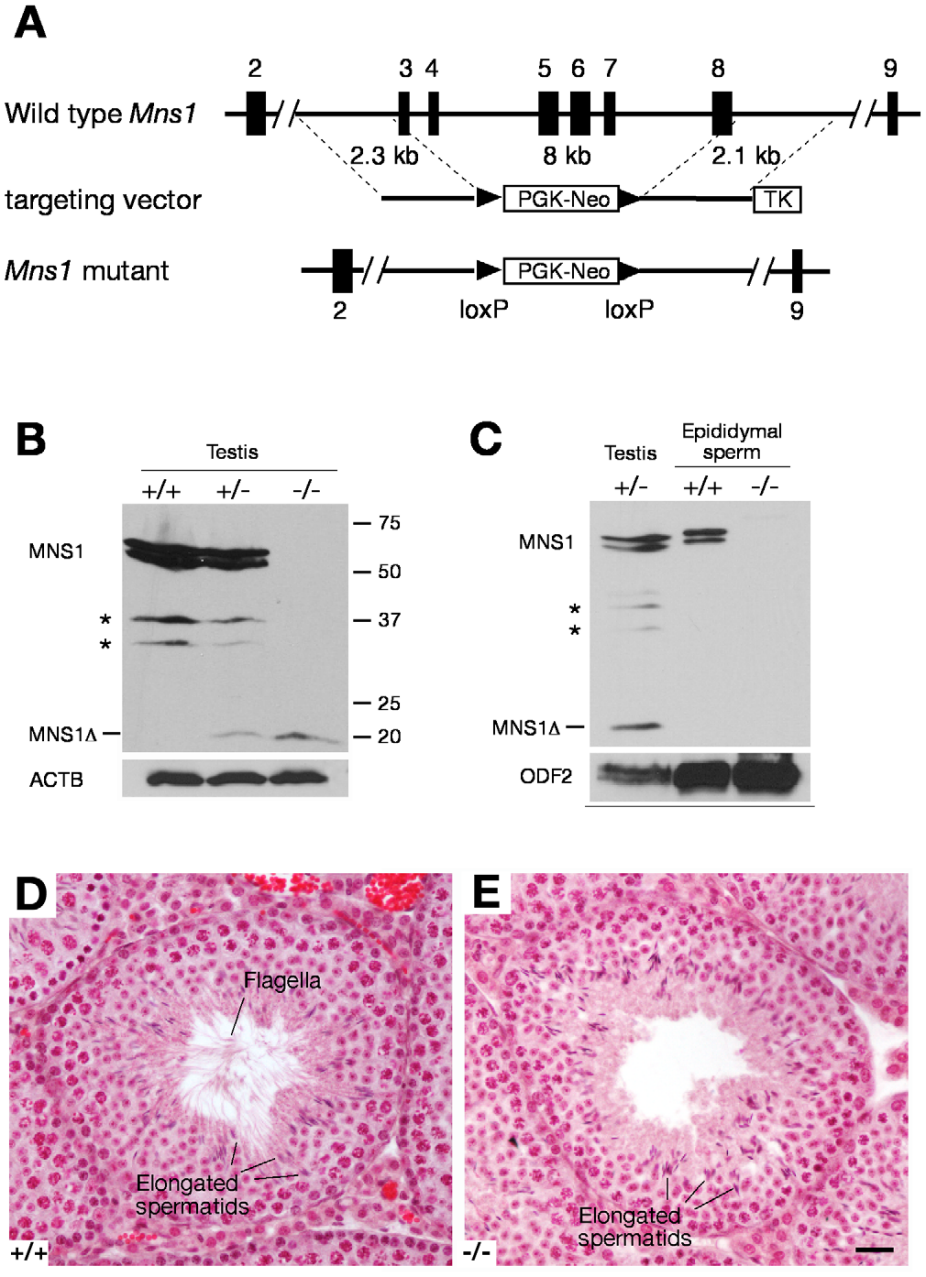
*Mns1* is required for spermiogenesis. (A) Targeted inactivation of the *Mns1* gene. Mouse *Mns1* (encoding a protein of 491 aa) is a 10-exon gene spanning over a 20-kb genomic region on Chr. 9. The targeting strategy was to delete exons 3–8 (8-kb genomic region) by replacing it with the PGK-Neo selection cassette. Deletion of exons 3–8 removed aa 76–423. (B) Western blot analysis of 8-wk-old wild type, *Mns1* ^+/−^, and *Mns1* ^−/−^ testes. The mutant MNS1 protein (143 aa; MNS1Δ) is indicated. Asterisks designate the two intermediate MNS1 protein species. Anti-MNS1 antibody UP2060 was used. ACTB served as a control. (C) Western blot analysis of epididymal sperm. The two intermediate MNS1 protein species were absent in epididymal sperm. Anti-MNS1 antibody UP2060 was used. ODF2 served as a control. (D and E) Histological analysis of testes from adult wild type and *Mns1* ^−/−^ mice. The flagella were abundant in wild type seminiferous tubules (D) but were absent in *Mns1*-deificient tubules (E). Scale bar, 25 µm.

To determine the requirement of *Mns1* for spermatogenesis, we generated *Mns1* mutant mice by homologous recombination in embryonic stem (ES) cells (Figure 2A). Mouse *Mns1* (encoding a protein of 491 aa) is a 10-exon gene spanning over a 20-kb genomic region on Chr. 9. In the *Mns1* mutant, deletion of exons 3–8 removes aa 76–423 (Figure 2A). As expected, the internally deleted MNS1 protein (143 aa, MNS1Δ) was expressed in *Mns1* ^+/−^ and *Mns1* ^−/−^ testes (Figure 2B). Western blot analysis revealed two additional MNS1 cross-reactive bands (∼32–37 kD) in the testis (Figure 2B). These two intermediate bands were likely to be specific to MNS1, since they were not detected in *Mns1* ^−/−^ testes (Figure 2B). However, neither the two intermediate protein species nor MNS1Δ was detected in mutant sperm, suggesting that they are not incorporated into the flagella (Figure 2C).

Interbreeding of heterozygous mice yielded slightly fewer homozygous *Mns1* mutant offspring (*Mns1* ^+/+^, *Mns1* ^+/−^, *Mns1* ^−/−^: 73, 155, 50) (χ^2^ = 7.49, *P* = 0.024), suggesting lethality in some *Mns1*-deficient embryos or pups. However, *Mns1* ^−/−^ mice grew to adulthood with no gross abnormalities and no increased lethality was observed in *Mns1* ^−/−^ mice after weaning. The body weight of *Mns1* ^−/−^ adult mice was not different from that of wild type animals. Notably, *Mns1* ^−/−^ males were sterile, whereas *Mns1* ^−/−^ females were fertile. The weight of *Mns1* ^−/−^ testes (158±18 mg per pair) from 8-wk-old mice was slightly lower (13%) than that of the wild type (182±10 mg per pair) (Student's *t* test, *P*<0.019). Strikingly, the sperm count from *Mns1* ^−/−^ epididymides (0.77×10^6^ per cauda) was dramatically reduced to only 8% of the wild type level (9.4×10^6^ per cauda) (*P*<0.0001). The *Mns1*-deficient epididymides were mostly filled with cell debris and contained much fewer sperm (Figure S2). Histological analysis revealed that seminiferous tubules from adult *Mns1* ^−/−^ testes contained all stages of germ cells but the mutant elongated spermatids appeared to lack flagella, in comparison with wild type elongated spermatids (Figure 2D, 2E). Thus, MNS1 is required for male fertility and is essential for spermiogenesis.

### MNS1 Is Required for the Assembly of Sperm Flagella

**Figure 3:**
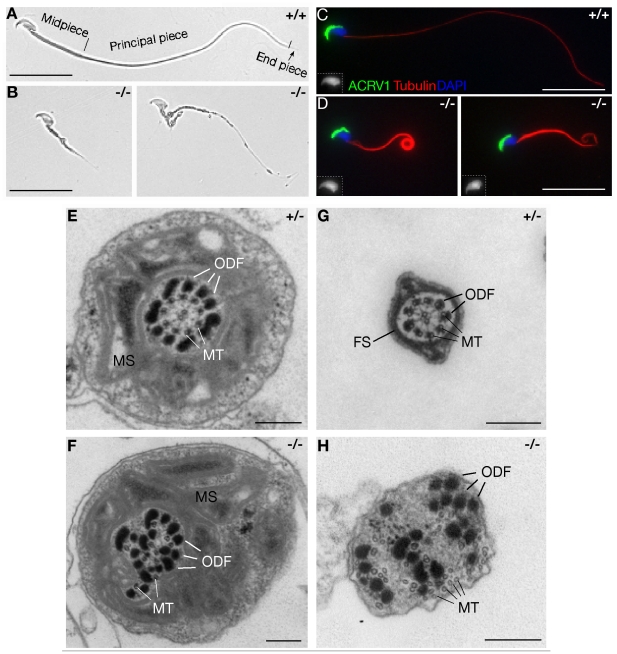
MNS1 is essential for the assembly of sperm flagella. (A) Wild type epididymal sperm. Midpiece, principal piece and end piece of flagella are indicated. (B) Defective flagellar formation in *Mns1*-deficient epididymal sperm. (C and D) Immunofluorescence analysis of wild type (C) and *Mns1*-deficient sperm (D). Sperm nuclei are shown in the insets. (E–H) Ultrastructural analysis of testicular sperm flagella. (E, F) The midpiece of sperm from *Mns1* ^+/−^ and *Mns1* ^−/−^ testes. (G, H) The principal piece of sperm from *Mns1* ^+/−^ and *Mns1* ^−/−^ testes. FS, fibrous sheath; MS, mitochondria sheath; MT, microtubules; ODF, outer dense fibers. Scale bars, 25 µm (A–D) and 250 nm (E–H).

We next analyzed the sperm from the cauda epididymis. The sperm flagellum is divided into the midpiece, principal piece, and the short end piece (Figure 3A). We found that the great majority (98%) of sperm from *Mns1* ^−/−^ mice have a very short crooked tail (less than 25% the wild type length) (Figure 3A, 3B). Rarely, sperm with a long flagellum was observed but its flagellum appeared to be abnormal (Figure 3B). In addition to abnormal flagellar morphology, freshly isolated MNS1-deficient sperm exhibited no motility upon microscopic examination. However, the head of MNS1-deficient sperm appeared normal in morphology (Figure 3D, inset) and contained protamine 1 (Figure S3), suggesting that nuclear elongation and nuclear condensation events were not disrupted during spermiogenesis in MNS1-deficient mice. Acrosomes in MNS1-deficient sperm also appeared to be normal in morphology (Figure 3D).

Morphological analysis revealed abnormal assembly of sperm flagella in *Mns1* ^−/−^ mice. Immunofluorescence microscopy showed that MNS1-deficient sperm flagella were positive for tubulin, demonstrating the presence of microtubules (Figure 3D). In addition to the “9+2” microtubular axoneme and outer dense fibers (ODFs) of the wild type flagella, the midpiece and principal piece consist of mitochondrial sheath and fibrous sheath respectively (Figure 3E, 3G). In the MNS1-deficient flagella, the axonemal microtubules and ODFs were completely disorganized (Figure 3F, 3H). The mitochondrial sheath did form but in uneven thickness around the axoneme in the midpiece of the mutant sperm (Figure 3F). The fibrous sheath appeared to be missing in the principal piece of MNS1-deficient sperm (Figure 3H). These analyses demonstrated that MNS1 is essential for the assembly of sperm flagella in mice.

### Randomized Left–Right Asymmetry and Hydrocephalus in MNS1–Deficient Mice

**Figure 4:**
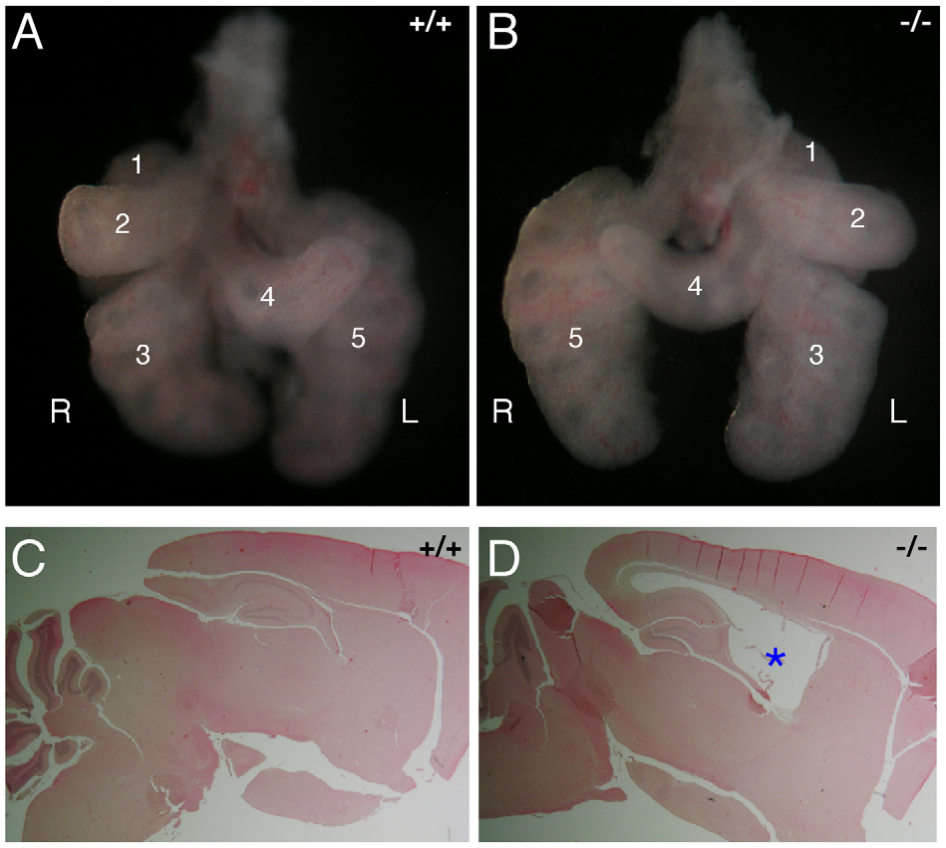
Abnormal left–right patterning and hydrocephalus in *Mns1*-deficient mice. (A, B) Lung from wild type E12.5 embryos displayed situs solitus (A), whereas lung from 36% of *Mns1* ^−/−^ embryos exhibited situs inversus (B). The five lung lobes were labeled for orientation purpose. (C, D) Sagittal sections of brain from post-natal day 24 wild type mouse (C) and *Mns1* ^−/−^ (D) mouse. The asterisk denotes the enlarged ventricle (hydrocephalus) in the *Mns1* ^−/−^ brain.

We next tested whether MNS1 is required for somatic ciliary functions. Motile cilia such as ependymal cilia and nodal cilia, also consist of axonemes. Nodal cilia are required for left-right patterning of internal organs during embryogenesis and nodal ciliary dysfunction causes situs inversus [11], [12]. Ependymal cilia are responsible for the flow of cerebrospinal fluid and their defects lead to hydrocephalus [8]–[10]. We found that *Mns1* ^−/−^ mice exhibited abnormal left-right asymmetry. Based on lung lobation patterns, all 13 wild type and 34 *Mns1* ^+/−^ embryos exhibited normal left-right patterning (Figure 4A). However, eight of 22 *Mns1* ^−/−^ embryos examined (E12.5–E16.5) displayed normal situs, eight presented with situs inversus (Figure 4B), and six exhibited left isomerism, indicating that MNS1 is required for nodal ciliary function. In contrast with wild type mice (Figure 4C), post-natal day 24-old *Mns1* ^−/−^ mice developed hydrocephalus (Figure 4D), suggesting that MNS1 is also important for ependymal ciliary function. By histological analysis of brain from mice at post-natal days 2, 4, and 16, we found that *Mns1*-deficient mice exhibited no hydrocephalus at post-natal day 2 but began to develop hydrocephalus at post-natal day 4. Taken together, these data have shown that MNS1 is essential for motile ciliary functions.

Non-motile cilia are thought to be involved in sensory perception and thus are important for signaling processes such as Hedgehog and Wnt signaling pathways [24], [25]. Defects in non-motile ciliary functions cause polycystic kidney disease, polydactyly, retinal degeneration, etc [5], [6]. However, the MNS1-deficient mice lacked polycystic kidney disease and polydactyly, suggesting that MNS1 is not required for non-motile ciliary functions.

### Defects in Outer Dynein Arms in MNS1–Deficient Tracheal Motile Cilia

**Figure 5:**
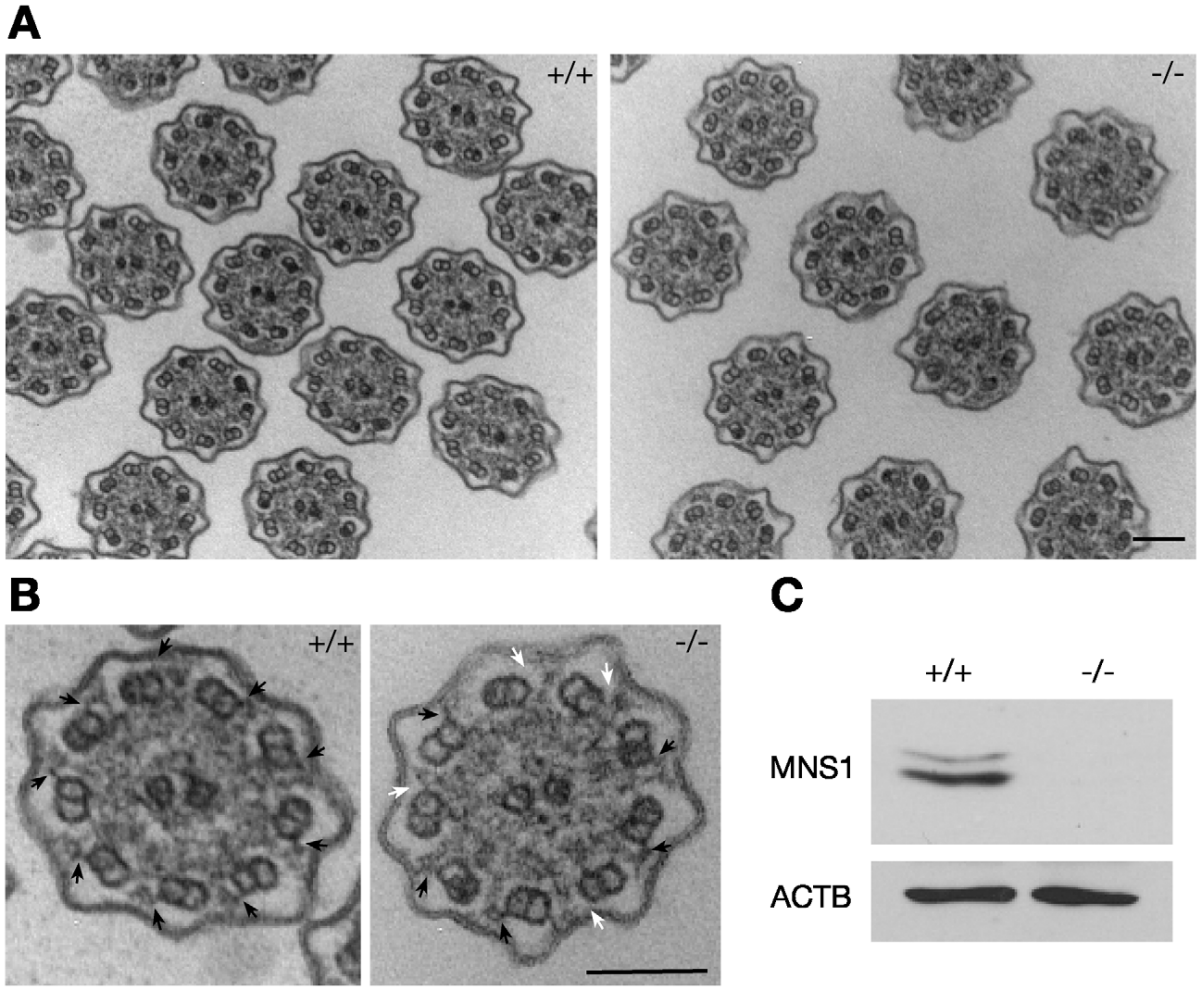
Defects in outer dynein arms in *Mns1*-deficient tracheal cilia. (A) Ultrastructural analysis of cross sections of tracheal cilia (low magnification). (B) Ultrastructural analysis of tracheal cilia (high magnification). Solid arrows indicate the presence of outer dynein arms, and white arrows indicate positions of missing outer dynein arms. (C) Western blot analysis of MNS1 in adult trachea (Antibody UP2284). ACTB served as a control. Scale bars, 100 nm.

Next we performed ultrastructural analysis of tracheal motile cilia. Unlike the disorganized axonemal structure in MNS1-deficient sperm flagella (Figure 3), the characteristic “9+2” axonemal structure was present in MNS1-deficient tracheal cilia (Figure 5A, 5B). However, close examination revealed that MNS1-deficient motile cilia lack some outer dynein arms and contain only ∼4 of them (3.8±1.6, n = 24) per cross section, in contrast with the 9 outer dynein arms in the wild type (Figure 5B), suggesting that MNS1 is an axonemal protein. MNS1 was previously identified in the ciliary proteome of human bronchial epithelial cells [26]. Our Western blot analysis showed that MNS1 protein is expressed in wild type trachea but absent in *Mns1* ^−/−^ trachea (Figure 5C).

### MNS1 Monomers Interact with Each Other

**Figure 6:**
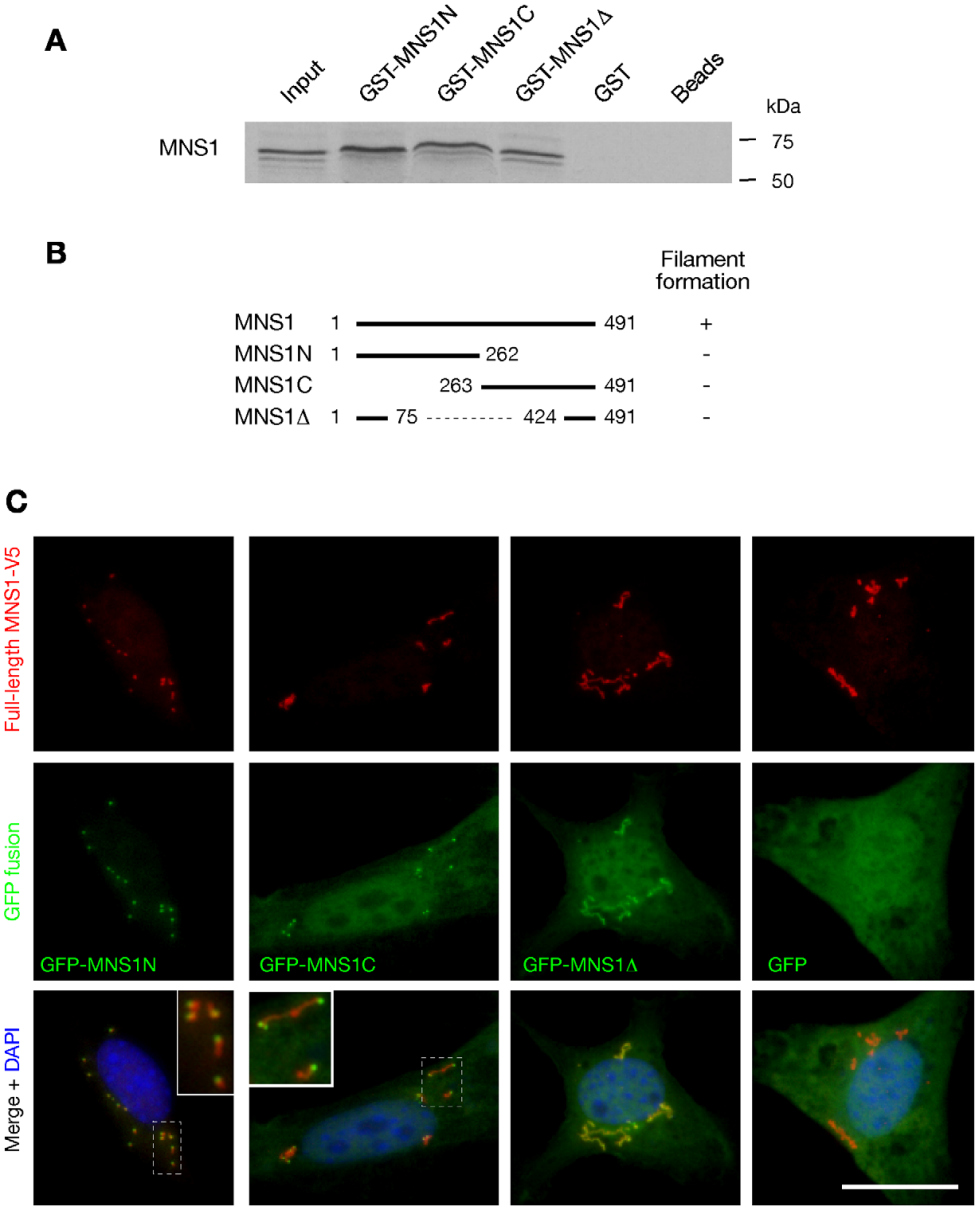
MNS1 interacts with itself. (A) GST-pulldown assay. GST fusion proteins MNS1N, MNS1C, and MNS1Δ were expressed in *E. coli*. Full-length MNS1 was in vitro translated in the presence of [^35^S]methionine. GST and beads alone served as controls. (B) Only full-length MNS1 formed filaments in NIH 3T3 cells. MNS1Δ is the internally-deleted mutant protein in the *Mns1* ^−/−^ testes. The numbers next to the endpoints indicate the corresponding amino acid positions. (C) Localization of truncated MNS1 fragments to the MNS1 filaments in NIH 3T3 cells. The full-length MNS1 was tagged with the V5 epitope. Truncated MNS1 proteins were fused with GFP. Both MNS1N and MNS1C localized to one end of MNS1 filaments (inset: enlarged view). Scale bar, 25 µm.

To elucidate a potential molecular mechanism for the function of MNS1 in spermiogenesis and motile ciliary functions, we performed a yeast two-hybrid screen of a human testis cDNA library using the full-length human MNS1 as bait. In this screen, we identified clones encoding the C-terminal half of MNS1. We confirmed the self-interaction of mouse MNS1 by yeast two-hybrid assay (data not shown). We then performed GST pulldown experiments to test if MNS1 physically binds to itself. These in vitro experiments showed that MNS1 binds to GST-MNS1N, GST-MNS1C. and GST-MNS1Δ, but not GST alone, indicating that MNS1 might form dimers or oligomers (Figure 6A). The self-interaction of MNS1 could be mediated by its coiled-coil domain, as both MNS1N and MNS1C contain coiled coil domains. We then analyzed the secondary structure of MNS1Δ (143 aa), which consists of the N-terminal 75 residues and the C-terminal 68 residues (Figure 6B). MNS1Δ was predicted to harbor a coiled coil domain in its middle region, which spans residues from both fragments. Therefore, the coiled coil domain in MNS1Δ may mediate its interaction with the full-length MNS1.

To determine whether MNS1 is able to form oligomers, we expressed MNS1 in NIH 3T3 fibroblast cells. Our data suggest that the full-length MNS1 protein forms short filaments in the cytoplasm (Figure 6B and Figure S4A). In contrast, the three MNS1 truncated proteins (MNS1N, MNS1C, or MNS1Δ) localized diffusely throughout the cells and did not form filaments (Figure 6B and Figure S4B–S4D). These data suggest that only the full-length MNS1 protein is capable of forming polymers. Although over-expression of the full length MNS1 may form non-specific aggregates rather than organized polymers in this assay, we favor the interpretation that MNS1 forms filaments. However, additional electron microscopic and biochemical studies are required to fully understand the structural basis.

Using this filament formation assay, we then tested if the three truncated MNS1 proteins would incorporate into the full-length MNS1 structures (Figure 6B). We co-expressed the full-length MNS1 and each of the three truncated MNS1-GFP fusion proteins in NIH 3T3 cells (Figure 6C). Consistent with our yeast two-hybrid interaction and GST pulldown data, MNS1C localized to the MNS1 filaments, remarkably, to one end of the filaments, suggesting the polarity of the MNS1 filaments (Figure 6C). Interestingly, MNS1N also localized to one end of MNS1 filaments. In contrast, MNS1Δ localized continuously along the MNS1 filaments (Figure 6C), while GFP-MNS1Δ alone did not from aggregates (Figure S5). These data showed that all three MNS1 mutant proteins interact with the full-length MNS1 protein. Importantly, these data suggested that MNS1 filaments possess a polarity and that MNS1 may be assembled in a head to tail fashion.

## Discussion

Here we report that MNS1 is essential for spermiogenesis but dispensable for meiosis. In a previous study, it was concluded that MNS1 is specifically expressed in pachytene spermatocytes [20]. However, we found that the MNS1 protein is abundantly expressed in late pachytene spermatocytes, diplotene spermatocytes, and spermatids and thus is not restricted to pachytene spermatocytes. Using different MNS1 antibodies that we generated, we always detected MNS1 as two closely migrating bands (∼60 kD) in protein extracts from testis, epididymal sperm, and trachea. Our antibodies were specific for MNS1, as evidenced by the absence of two ∼60 kD bands in MNS1-deficient testes, sperm, and trachea. However, in the previous study, the “MNS1” antibody recognized only one 60 kD band in testes and did not recognize any 60 kD protein in epididymal sperm by Western blotting analyses, raising the possibility that the antibody generated in that study might not be specific to MNS1 [20].

Several lines of evidence support that MNS1 plays an essential role in the assembly of sperm flagella. Firstly, MNS1 is an integral component of sperm flagella. The localization of MNS1 to SDS-treated sperm flagella suggests that MNS1 is an SDS-resistant component of sperm flagella. Secondly, MNS1 monomers interact with each other and are able to form fibrous polymers when ectopically expressed, implying that MNS1 might be assembled into filamentous structures in the flagella. Thirdly, in the absence of MNS1, sperm flagella are very short. Microtubules and ODFs are completely disorganized in the mutant sperm.

Inactivation of MEIG1, PACRG, or SPEF2 causes disorganization of microtubules and ODFs in sperm flagella, a phenotype similar to that in MNS1-deficient mice [27]–[30]. MEIG1 and PACRG interact with each other and both are expressed in spermatids [27], [29]. PACRG localizes to the postacrosomal region of the sperm head and the midpiece of the flagellum [29]. Disruption of SPEF2 by a LINE1 retrotransposon insertion in boars and inactivation of SPEF2 in mice led to immotile short-tail sperm defect [10], [31]. However, our yeast two-hybrid screen using MNS1 as bait did not detect any of these proteins. A proteomic screen has identified ∼50 proteins, including MNS1, from the accessory structures of mouse sperm flagellum [22]. These genetic and proteomic studies underscore the complexity in the structure and assembly of sperm flagella.

Based on the NCBI database search, MNS1 is conserved only in organisms with motile cilia. According to the ciliome database [5], MNS1 homologues were identified in a number of genomic and proteomic studies of ciliary proteins, for example, in *Chlamydomonas reinhardtii* [32]–[34], *C. elegans* [35], [36], and in humans [26], [37]. Therefore, in addition to male sterility, MNS1-deficient mice exhibit situs inversus and hydrocephalus, suggesting an essential role for MNS1 in motile cilia function and implicating MNS1 in primary ciliary dyskinesia (PCD). Interestingly, MNS1-deficient tracheal motile cilia lack some outer dynein arms, suggesting that MNS1 is an axonemal protein in the motile cilia. A number of genes are implicated in human, dog, and mouse PCD, including *DNAI1* [13], *DNAH11* [17], *DNAH5* [18], [19], *TXNDC3* [38], *Spag6* [9], *Pcdp1* [16], *CCDC39* [15], *CCDC40* [14], *Spef2* [10], etc. However, MNS1-deficient mice grew to adulthood, suggesting that hydrocephalus is relatively mild in these mutant mice. Therefore, our study of MNS1 has important implications for male infertility in humans. Our findings on the function of MNS1 from mouse studies will help to select a cohort of infertile men with defined phenotypes for mutation screening in the human *MNS1* gene.

## Materials and Methods

### Ethics Statement

Mice were maintained and used for experimentation according to the guidelines of the Institutional Animal Care and Use Committee of the University of Pennsylvania.

### Generation of MNS1 Antibodies

Two GST-mouse MNS1 (amino acids 1–233 and 392–491) fusion proteins were expressed in *Escherichia coli* using the pGEX4T-1 vector and affinity purified with glutathione Sepharose. Purified recombinant proteins were used to immunize rabbits at Cocalico Biologicals Inc, resulting in MNS1 antiserum UP2060 against GST-MNS1 (aa 1–233) and UP2284 against GST-MNS1 (aa 392–491). Specific antibodies were affinity purified with the immunoblot method as previously described [39].

### SDS-EDTA Treatment of Sperm and Western Blot Analysis

The epididymal sperm were collected from 3 male mice (C57BL/6) by squeezing the caudal epididymides in 1xPBS solution and centrifugation at 800 g for 5 min at RT. Sperm were homogenized in 1 ml of SDS-EDTA solution (1% SDS, 75 mM NaCl, 24 mM EDTA, pH 6.0) and centrifuged at 5000 g for 30 min at RT. 100 µl of SDS-PAGE sample buffer (62.5 mM Tris, pH 6.8, 3% SDS, 10% glycerol, 5% β-mercaptoethanol, 0.02% bromophenol blue) was added to 100 µl of supernatant, while the pellet was resuspended in 200 µl of SDS-PAGE sample buffer. The samples were heated at 95°C for 10 min and 20 µl of each sample was used for western blot analysis.

### Targeted Disruption of the *Mns1* Gene

To generate the *Mns1* targeting construct, DNA fragments were amplified by high-fidelity PCR using an *Mns1* BAC clone (RP23-349N4) as template (Figure 2A). V6.5 ES cells were electroporated with linearized targeting construct (pUP101/ClaI). Screening of ES cells was described previously and revealed a targeting frequency of 11% [40]. Two homologously targeted ES cell clones (B6 and G5) were injected into B6C3F1 (Taconic) blastocysts. The *Mns1* mutant allele was transmitted through the germline in chimeric mice derived from both clones. All offspring were genotyped by PCR. Wild-type allele (500 bp) was assayed by PCR with the primers GTCAGGAAGATCTACGAGGA and CCAGAAGTCTTGTGCCCTCT. The *Mns1* knockout (300 bp) allele was assayed by PCR with the primers GTCAGTTTGCTGTTGTAGAGT and CCTACCGGTGGATGTGGAATGTGTG.

### Yeast Two-Hybrid Screen and GST-Pulldown Assay

A pretransformed Mate & Plate human testis cDNA library (Clontech) was screened using the full-length human MNS1 cloned into pGBKT7 as bait. GST-pulldown experiments were performed as previously described [41].

### Histology, Immunofluorescence, and Western Blot Analyses

For histology, testes were fixed in Bouin's solution and brains were fixed in 4% PFA overnight. Samples were processed, sectioned, and stained with hematoxylin and eosin. For immunofluorescence analysis, testes were fixed in 4% PFA for 3 h at 4°C. Epididymal sperm and NIH 3T3 cells on slides were fixed with 4% PFA for 5 min at RT. Immunofluorescence was performed as previously described [42].

For Western blot analyses, adult tissues were homogenized in a glass homogenizer in SDS-PAGE sample buffer and heated at 95°C for 10 min. Protein concentration was measured using the Bradford assay. 30 µg of total protein for each sample was resolved on 10% SDS-PAGE gel and electro-blotted onto PVDF membrane. Primary antibodies used were MNS1 (this study), ODF2 (Santa Cruz), ACRV1 (P. P. Reddi) [43], Protamine 1 (SHAL) [44], β-tubulin (DSHB), ACTB (Sigma-Aldrich). Texas Red or FITC-conjugated secondary antibodies were used for immunofluorescence. HRP-conjugated secondary antibodies were used for Western blot analyses.

### Electron Microscopy (EM)

EM was performed at the Biomedical Imaging Core facility at the University of Pennsylvania. Adult testes and dissected trachea were fixed in 2.5% glutaraldehyde and 2% paraformaldehyde overnight, and postfixed in 1% osmium tetroxide for 1 hour. The specimens were further processed and sectioned at the core facility as previously described [40].

### Expression Constructs and Cell Culture

The cDNAs encoding full-length or truncated MNS1 were cloned into pcDNA3.1/V5-His TOPO TA for V5 tagging, or pEGFP-C1 for GFP fusion. Transfection of NIH 3T3 cells was performed using the Lipofectamine reagent (Invitrogen). 24–48 hours after transfection, cells were washed with PBS, fixed in PFA, stained with anti-V5 antibody (Invitrogen), incubated with secondary antibodies, mounted with DAPI-containing Vectashield mounting medium (Vector Labs) and visualized on a Zeiss Axioskop 40 microscope with a digital imaging system.

## Supporting Information

Figure S1 Expression of MNS1 in mouse spermatogenesis. Frozen sections of testes from adult wild type mice were immunostained with anti-MNS1 antibody (UP2060) (top panels). Nuclear morphology of germ cells (DAPI, bottom panels) was used to determine the stages of seminiferous tubules. The stage numbers are shown in Roman numerals. Abbreviations: Sg, spermatogonium; Pl, preleptotene spermatocyte; Lp, leptotene spermatocyte; Zg, zygotene spermatocyte; Pa, pachytene spermatocyte; Dp, diplotene spermatocyte; RS, round spermatid; ES, elongating spermatid. Scale bar, 25 µm.
(TIFF)

Figure S2 Histology of epididymides from adult mice. While wild type epididymal tubules (A) were full of mature sperm, epididymal tubules from *Mns1*-deficient mice (B) were filled with cell debris and contained much fewer sperm. Scale bar, 50 µm.
(TIFF)

Figure S3 Immunofluorescence analysis of sperm nuclei with anti-Protamine 1 antibody. Epididymal sperm from *Mns1* ^+/−^ (A, A') and *Mns1* ^−/−^ (B, B') mice were used. Panels A and B are bright field images. Anti-protamine 1 antibody was purchased from SHAL Technologies (Hup1N).
(TIFF)

Figure S4 Distribution of the full-length and truncated MNS1 proteins in NIH 3T3 cells. All the proteins were tagged with the V5 epitope. Immunofluorescence was performed with anti-V5 monoclonal antibodies. DNA was stained with DAPI. Scale bar, 10 µm.
(TIFF)

Figure S5 Distribution of GFP-MNS1Δ in NIH 3T3 cells. The fluorescence of GFP-MNS1Δ was directly observed after transfection of NIH 3T3 cells. DNA was stained with DAPI. Scale bar, 25 µm.
(TIFF)
