# Supplementary figures and images for: MNS1 Is Essential for Spermiogenesis and Motile Ciliary Functions in Mice
Source: PLoS Genet. 2012 Mar 1;8(3):e1002516. doi: 10.1371/journal.pgen.1002516 (PMC3291534; doi:10.1371/journal.pgen.1002516)

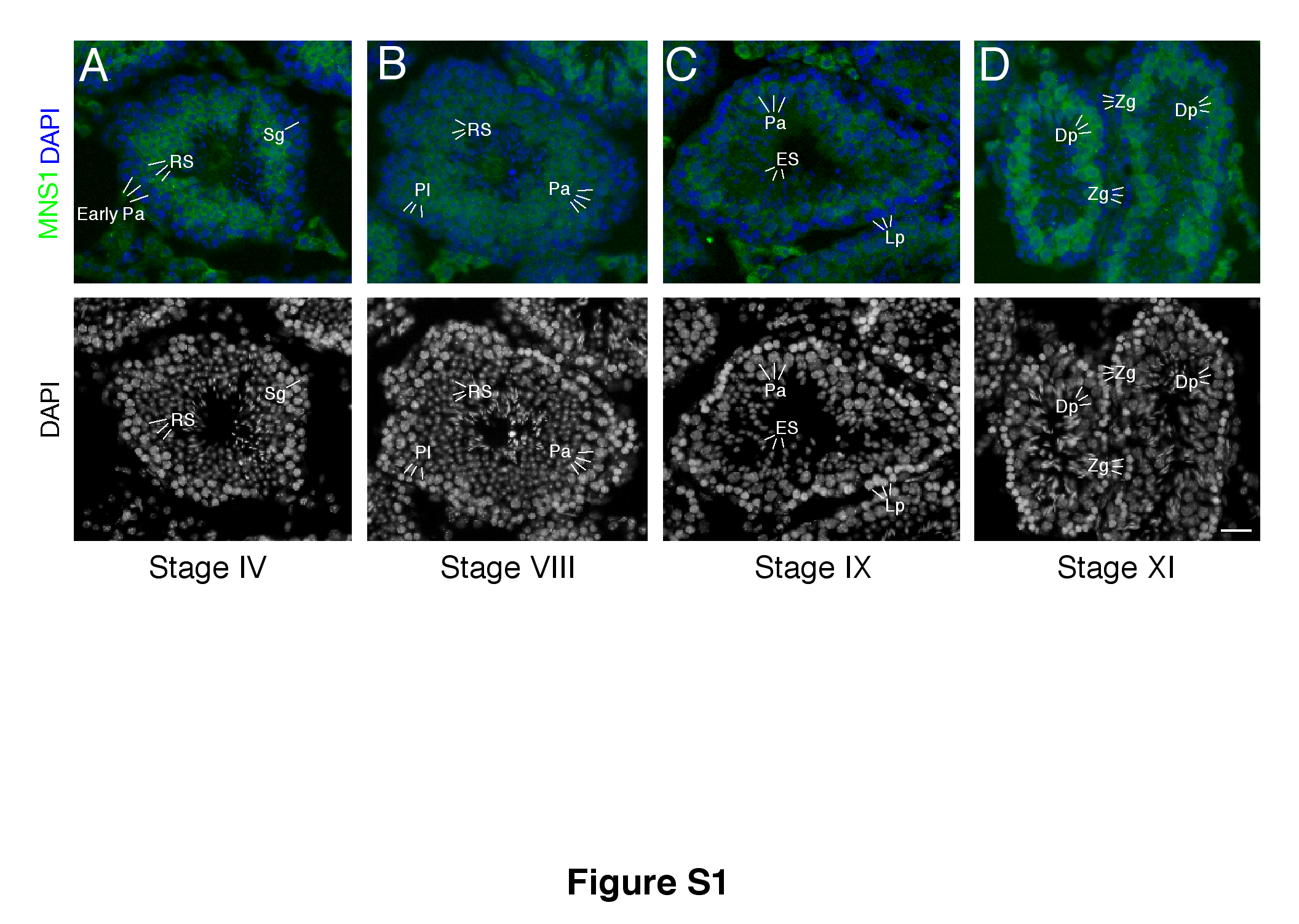

Supplement: Figure S1 — Expression of MNS1 in mouse spermatogenesis. Frozen sections of testes from adult wild type mice were immunostained with anti-MNS1 antibody (UP2060) (top panels). Nuclear morphology of germ cells (DAPI, bottom panels) was used to determine the stages of seminiferous tubules. The stage numbers are shown in Roman numerals. Abbreviations: Sg, spermatogonium; Pl, preleptotene spermatocyte; Lp, leptotene spermatocyte; Zg, zygotene spermatocyte; Pa, pachytene spermatocyte; Dp, diplotene spermatocyte; RS, round spermatid; ES, elongating spermatid. Scale bar, 25 µm. (TIFF) [file pgen.1002516.s001.tiff]

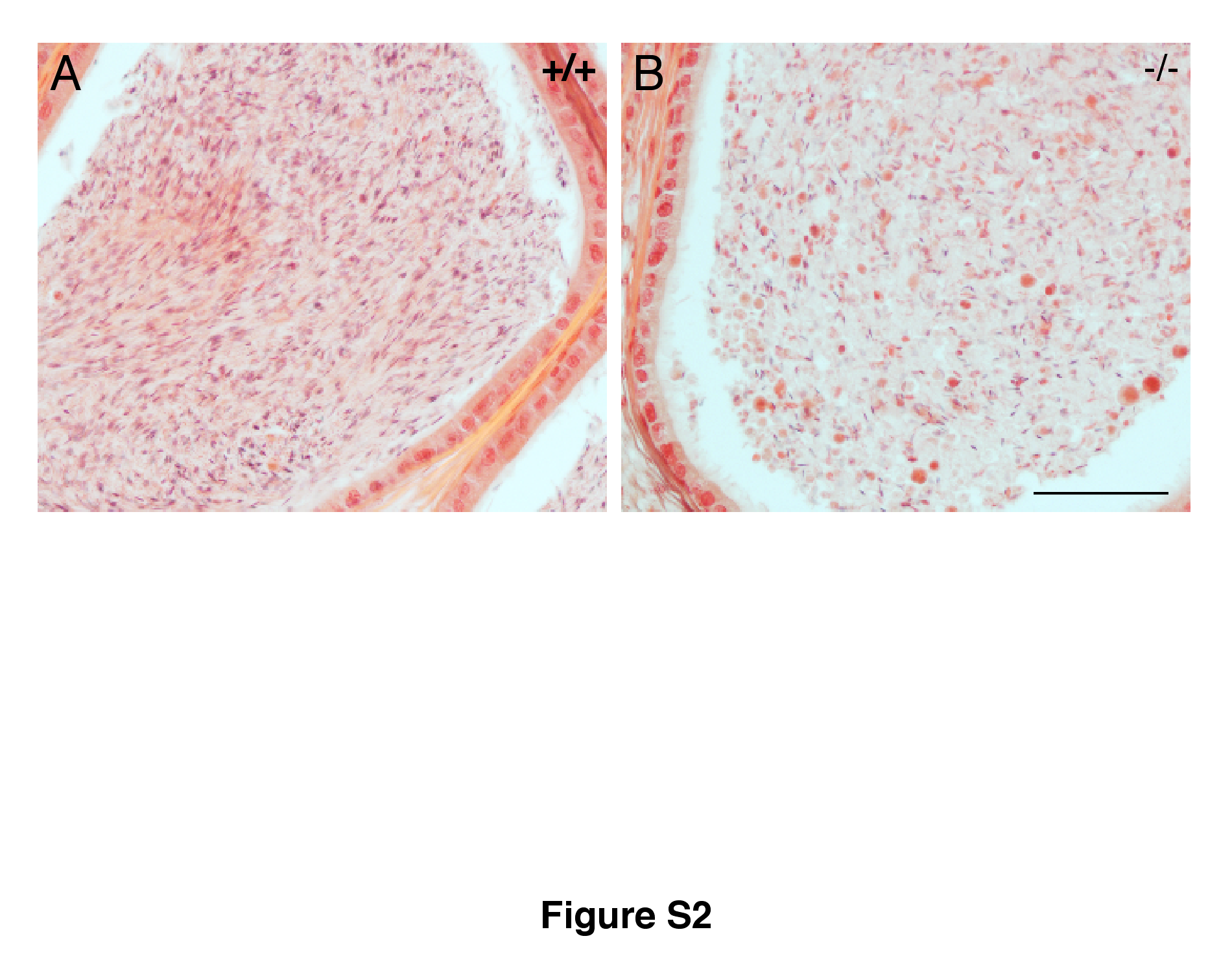

Supplement: Figure S2 — Histology of epididymides from adult mice. While wild type epididymal tubules (A) were full of mature sperm, epididymal tubules from Mns1-deficient mice (B) were filled with cell debris and contained much fewer sperm. Scale bar, 50 µm. (TIFF) [file pgen.1002516.s002.tiff]

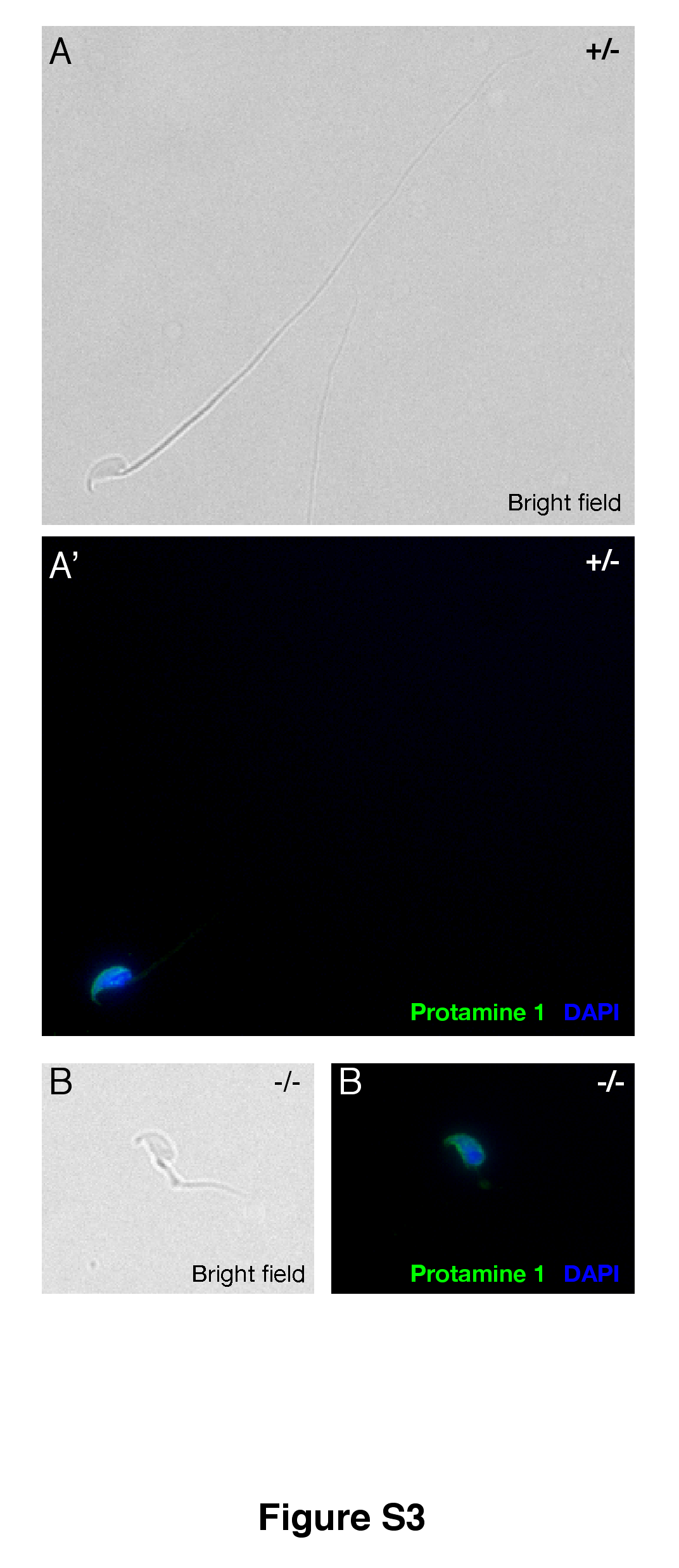

Supplement: Figure S3 — Immunofluorescence analysis of sperm nuclei with anti-Protamine 1 antibody. Epididymal sperm from Mns1 +/− (A, A') and Mns1 −/− (B, B') mice were used. Panels A and B are bright field images. Anti-protamine 1 antibody was purchased from SHAL Technologies (Hup1N). (TIFF) [file pgen.1002516.s003.tiff]

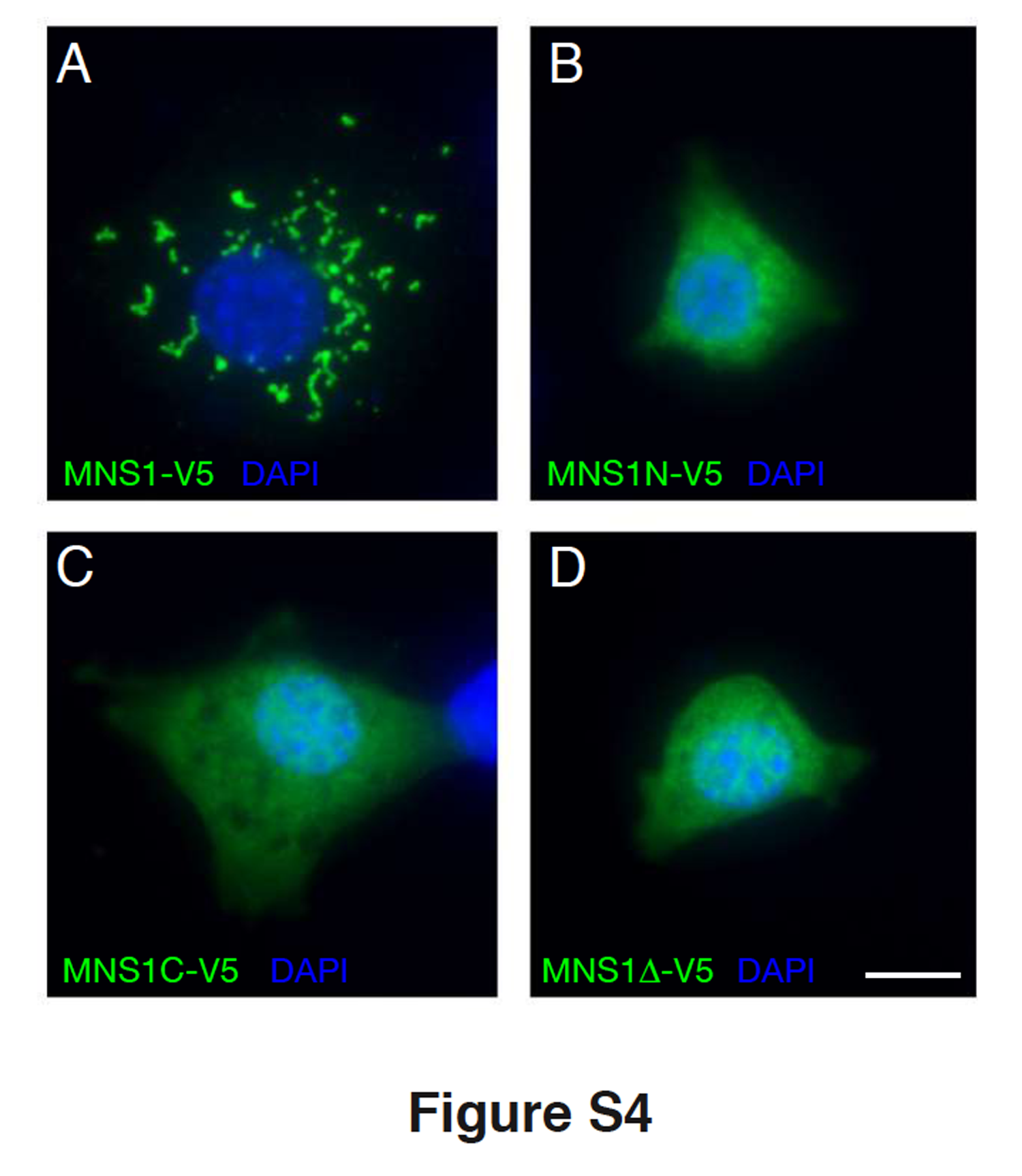

Supplement: Figure S4 — Distribution of the full-length and truncated MNS1 proteins in NIH 3T3 cells. All the proteins were tagged with the V5 epitope. Immunofluorescence was performed with anti-V5 monoclonal antibodies. DNA was stained with DAPI. Scale bar, 10 µm. (TIFF) [file pgen.1002516.s004.tiff]

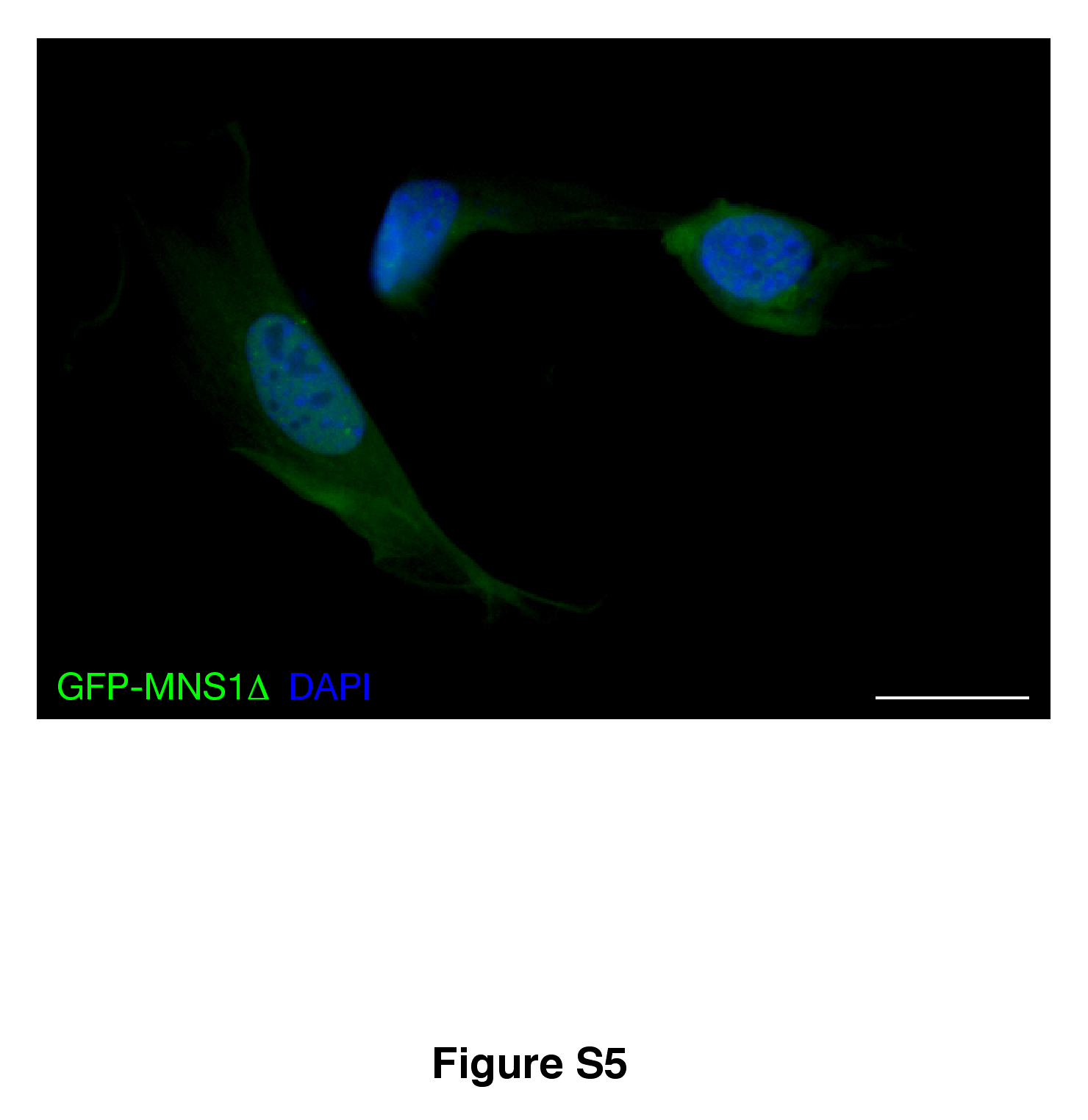

Supplement: Figure S5 — Distribution of GFP-MNS1Δ in NIH 3T3 cells. The fluorescence of GFP-MNS1Δ was directly observed after transfection of NIH 3T3 cells. DNA was stained with DAPI. Scale bar, 25 µm. (TIFF) [file pgen.1002516.s005.tiff]
